# Protection of neurons from high glucose-induced injury by deletion of MAD2B

**DOI:** 10.1111/jcmm.12229

**Published:** 2014-01-20

**Authors:** Xianfang Meng, Xiaolan Wang, Xiujuan Tian, Zhihua Yang, Man Li, Chun Zhang

**Affiliations:** a Department of Neurobiology, School of Basic Medical Sciences, Tongji Medical College, Huazhong University of Science and TechnologyWuhan, China; b Department of Nephrology, Union Hospital, Tongji Medical College, Huazhong University of Science and TechnologyWuhan, China

**Keywords:** MAD2B, hyperglycaemia, cyclin B1, neuronal injury

## Abstract

Diabetic encephalopathy may lead to cognitive deficits in diabetic patients and diminish quality of life. It has been shown that protracted hyperglycaemia is directly associated with neuronal apoptosis, which is involved in diabetic encephalopathy. The anaphase-promoting complex (APC) is essential for the survival of post-mitotic neurons. In our previous study, we found that the mitotic arrest deficient protein MAD2B, one of APC inhibitors, was expressed in neurons in central nervous system. However, whether MAD2B is involved in hyperglycaemia-induced apoptosis and thus takes part in diabetic encephalopathy is still unknown. To address this issue, we first explored the expression of MAD2B and cyclin B1 detected by immunofluorescence and Western blot. It was found that hyperglycaemia remarkably increased the expression of MAD2B and accumulation of cyclin B1 in cortices of diabetes mellitus rat model and in cultured primary neurons. To further explore the role of MAD2B in hyperglycaemia-induced neuronal injury, we depleted MAD2B expression by a specifically targeted shRNA against MAD2B. We observed that MAD2B deficiency alleviated cyclin B1 expression and apoptotic neuronal death. These results demonstrate that MAD2B expression is the main culprit for accumulation of cyclin B1 and apoptosis in neurons under high glucose. Moreover, inhibition of the expression of MAD2B prevented neurons from entering an aberrant S phase that led differentiated neurons into apoptotic cell death. These results suggest that hyperglycaemia induced neuronal apoptosis through inducing expression of MAD2B, which represents a novel mechanism of diabetic encephalopathy.

## Introduction

Diabetes mellitus (DM), or simply diabetes, is a group of metabolic diseases in which a person has high blood glucose. The number of people with diabetes is increasing for reasons of population growth, ageing, urbanization and increasing prevalence of obesity and physical inactivity. Diabetes mellitus is one of the most common chronic diseases in nearly all countries. Numerous epidemiological studies support a link between diabetes and Alzheimer*s disease, showing that diabetic patients over 65 years of age have a significantly increased risk of developing Alzheimer*s disease [1,2]. Experimental studies have also demonstrated that impaired performances on the Morris water maze and Y-maze are characteristic of streptozotocin (STZ)-induced diabetic rats [3,4]. These studies have demonstrated changes in brain function in diabetic patients and diabetic animal models, such as alterations in cognition, neuropsychology, neurobehaviour, electrophysiology, structure and neurochemistry. The type of central nervous damage caused by DM is also known as diabetic encephalopathy [5,6]. However, the molecular mechanisms by which diabetes impacts brain function and cognition are not fully understood.

Experimental studies have suggested that neuronal apoptosis may play a crucial role in neuronal loss and impaired cognitive function [7–9]. The involvement of neuronal apoptosis in diabetic encephalopathy has been demonstrated in diabetic animal models, and evidence of classical apoptosis was associated with decreased neuronal densities, and learning and cognitive deficits. Attenuation of apoptosis of neurons in the hippocampus and cerebral cortex ameliorated the cognition deficits of diabetic rats [8,10]. Moreover, neuronal injury and apoptosis in DM are directly associated with protracted hyperglycaemia [11]. However, the mechanisms of neuronal apoptosis in DM need to be explored.

Post-mitotic neurons, being terminally differentiated cells, generally do not enter the cell cycle after differentiation. Neurons undergo an active down-regulation of cell cycle-related proteins to survive. Recent studies demonstrated that aberrant cell-cycle activation plays a critical role in the brain of patients with neurodegenerative disorders. For example, the retinoblastoma protein family members are essential regulators of cell-cycle progression, principally through regulation of the E2f transcription factors. Growing evidence indicates that abnormal cell-cycle signals can participate in neuronal death [12]. Ectopic cell cycling occurs in both Alzheimer*s disease patients and mouse models, signifying that the loss of cell-cycle control is an important pathological root of the disease [13]. Moreover, cell-cycle disturbances emerge in the early stages of mild cognitive impairment and persist as the patient*s condition worsens [14]. Therefore, it is believed that aberrant activation of cell-cycle proteins plays a critical role in Alzheimer*s disease pathogenesis. The aberrant expression of proteins involved in the cell cycle has been also found in other neurodegenerative disorders such as Parkinson*s disease [15], amyotrophic lateral sclerosis [16] and Pick*s disease [17]. It has also been shown that aberrant activation of the cell-cycle machinery is thought to cause apoptosis in post-mitotic neurons in ischaemic models and in the brain of patients with focal brain infarction [18]. All these data suggest that after injury, some neurons in the brain re-enter the cell cycle and post-mitotic neurons generally undergo apoptosis when cell-cycle regulators are activated. However, whether cell-cycle proteins are involved in neuronal apoptosis in diabetic encephalopathy still needs to be elucidated.

The mitotic arrest deficient protein (MAD2B) is an important cell-cycle protein. The MAD2B gene, a human homologue of the Saccharomyces cerevisiae Rev7 gene, is located on the short arm of chromosome 1p36.16 [19]. It encodes the regulatory subunit of Pol ζ and has 53% similarity to a key mitotic spindle checkpoint protein, MAD2. In our previous study, we found that MAD2B can express in neurons of central nervous system (CNS) [20]. Whereas its role in mitotic spindle checkpoint control is well-documented, the exact role of MAD2B still remains to be established in neurons. Given the important role of cell-cycle proteins in neuronal apoptosis, the present study hypothesized that high glucose may induce neuronal injury and apoptosis through MAD2B pathway. To test the hypothesis, we first detected that whether high glucose induced MAD2B expression *in vivo* and *in vitro*. We then detected whether knocking down the expression of MAD2B alleviated high glucose-induced apoptosis.

## Materials and methods

### Ethics statement

All animal experimental procedures carried out in this study were approved by the Laboratory Animal of the Ethics Committee of Huazhong University of Science and Technology, and were in compliance with the guidelines for animal care set forth by this Committee.

### Induction of DM in rats

Diabetes mellitus with adult (250–280 g) male Sprague–Dawley rats was induced as we described previously [21]. Briefly, diabetes was induced by a 60 mg/kg intraperitoneal injection of STZ (60 mg/kg; Sigma-Aldrich, St. Louis, MO, USA) dissolved in sterile sodium citrate buffer solution (0.1 mol/l citric acid and 0.2 mol/l sodium phosphate, pH 4.5). Animals were considered diabetic if plasma glucose levels exceeded 16.7 mM [22], and the diabetic state was reconfirmed after 4 weeks prior to sacrifice.

### Primary neuronal cell culture and treatment

Primary neuronal cell culture and treatment with glucose were performed as described previously [20]. Briefly, primary cultures of rat cortical neurons were prepared from the of E17–E18 Sprague Dawley rat embryos. The cells were dissociated in Neurobasal medium (Gibco Invitrogen), supplemented with B27 (1:50 dilution; Gibco Invitrogen, Shanghai, China), 0.5 mM glutamine, 25 μM glutamate and 50 μg/ml gentamycin. The cells were plated in six-well plates coated with poly-d-lysine (0.1 mg/ml). The cultures were maintained in a humidified incubator with 5% CO_2_/95% air at 37°C for at least 6 days. This medium was subsequently given half-changes every 3 days. With the use of this protocol, over 95% of the cultured cells were neurons. When supplemented with B27, neurobasal medium is intended to give optimal growth and long-term survival to rat embryonic cortical neurons. Optimal survival rate and neurite growth of cortical neurons require 25 mM basal glucose, reflecting the fact that neurons have high metabolic rates. Neurobasal medium containing 25 mM glucose (control condition) meets these metabolic requirements and is essential for neuron culture. It has been reported that increasing the glucose concentration above 35 mM induces hyperglycaemic stress, ROS and cell injury [23]. Therefore, in the present study, we detected the expression of MAD2B at 37.5, 50 and 75 mM concentrations of glucose to produce a hyperglycaemic injury, which are similar to the ≥1.4-fold increase in blood glucose concentration in a person diagnosed as diabetic [24–26]. After 6 days in culture, half of the medium was replaced by fresh medium, and cells were incubated with different concentrations of glucose or mannitol (an osmotic control).

### Transduction of cortical neurons with lentiviral vectors

Cortical neurons were plated on poly-l-lysine-coated glass coverslips, and viral stocks were added after plating for 24 hrs. The lentiviral vectors express green fluorescent protein (GFP) and rat MAD2B shRNA was constructed by Genechem Co. Ltd. (Shanghai, China). According to the published paper [27], the shRNA sequence that targeted the rat MAD2B sequence (GenBank NM_001012106) was designed as follows: 5′-GAT GCA ACT TTA CGT AGA AGA. The randomly chosen nonsense sequence 5′-TTC TCG CAA CGT ATG CGC TGA-3′ was used as scramble control. After transfection for 5 days, the fluorescence of GFP was observed by using an excitation filter of 490 nm and an emission filter of 520 nm in a Nikon Diaphot inverted microscope equipped with a 75 W Xenon lamp and a Nikon 40X (Tokyo, Japan), 1.3 numerical aperture, epifluorescence oil immersion objective. Transfection efficiency was calculated by counting the number of fluorescein-positive cells *versus* the total number of cells in nine randomly selected regions from three independent experiments.

### RNA extraction and real-time RT-PCR

Total RNA was isolated from rat brain by using TRIzol reagent (Invitrogen, Shanghai, China) as described previously. The mRNA levels for target genes were analysed by real-time quantitative RT-PCR. The mRNAs for MAD2B and glyceraldehyde-3-phosphate dehydrogenase (GAPDH; internal control) were amplified and quantified with primers listed below. The synthetic oligonucleotide primer sequences for MAD2B and GAPDH were as follows: MAD2B 5′-TGC TTC GAG CCT TCA TTC TT-3′ (sense) and 5′-TGG ACA TCT TGC TCA TCT GC-3′ (antisense); GAPDH 5′-GGC ACA GTC AAG GCT GAG AAT G-3′ (sense) and 5′-ATG GTG GTG AAG ACG CCA GTA-3′ (antisense). Quantitative PCR was performed by using SYBR-Green dye (Applied Biosystems, Shanghai, China) and Applied Biosystems hardware and software (7500 RT-PCR System). Expression value of the targeted gene in a given sample was normalized to the corresponding expression of GAPDH. The 2^−ΔΔCt^ method was used to calculate relative expression of the targeted genes.

### Immunofluorescent staining

Primary antibody rabbit anti-MAD2B (1:300 dilution; Rockland Immunochemicals Inc., Gilbertsville, PA, USA), mouse anti-NeuN (1:50 dilution; Millipore Corporation, Billerica, MA, USA), rabbit anti-cyclin B1 (1:50 dilution; Proteintech Group, Wuhan, Hubei, China) was used in this study. After incubating the primary antibodies overnight at 4°C, the slides were incubated with different fluorescein-labelled secondary antibodies. Finally, the slides were mounted and subjected to examinations by using a confocal laser scanning microscope (Fluoview FV1000; Olympus, Tokyo, Japan).

### Western blot analysis

Western blot analyses were performed as previously described [28]. Primary antibodies to MAD2B (1:1000 dilution; Rockland Immunochemicals Inc.), cyclin B1 (1:1000 dilution; Cell Signaling Technology Inc., Danvers, MA, USA), cdc20 homologue (Cdh1, 1:300 dilution; Novus Biologicals, Littleton, CO, USA) and secondary antibodies horseradish peroxidase-labelled antimouse IgG or anti-rabbit IgG (1:6000 dilution; Santa Cruz Biotechnology, Santa Cruz, CA, USA) was used in this study. To document the loading controls, the membrane was reprobed with a primary antibody against housekeeping protein β-actin.

### TUNEL staining

According to the manufacturer*s instructions, Apoptosis was detected with the TMR red *In Situ Cell Death Detection Kit* (Roche, Mannheim, Germany). Terminal transferase was omitted as a negative control. Cells were exposed to DNase I prior to the assay (10 min.; Roche) to provide a positive control. TUNEL-positive cells were counted by an experimenter who was blind to the treatment groups.

### 5-ethynyl-2′-deoxyuridine (EdU) staining

The EdU is a nucleoside analogue of thymidine that is incorporated into DNA only during DNA synthesis allowing the visualization of newly synthesized DNA [29]. EdU staining was conducted by using EdU imaging kit (C00031, Apollo 567; RiboBio, Guangzhou, Guangdong, China) according to the manufacturer*s protocol. Briefly, after cells were treated with 50 mM glucose for 8 hrs, EdU was directly added to the culture medium at the final concentration 10 μM for another 16 hrs. Then cells were collected and washed with PBS. After being fixed in 4% paraformaldehyde and treated with 0.5% Triton-X for 15 min., cells were incubated in with Apollo, and nuclei were stained with Hoechst 33342.

### Statistics

Data are expressed as means ± SEM. The significance of the differences in mean values between and within multiple groups was examined by one-way anova followed by Duncan*s multiple range test. *P* < 0.05 was considered statistically significant.

## Results

### Hyperglycaemia induced MAD2B expression *in vivo* and *in vitro*

**Fig. 1:**
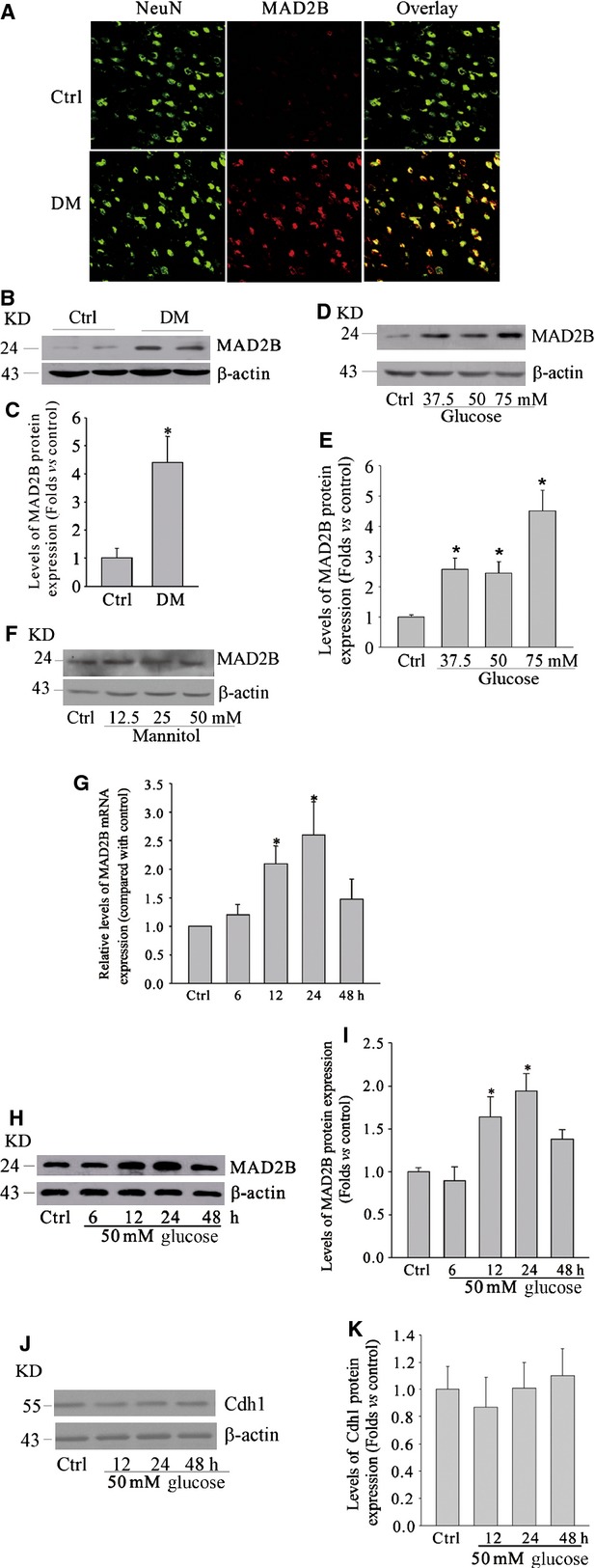
High glucose induced MAD2B expression *in vivo* and *in vitro*. (**A**) Representative confocal microscopic images of MAD2B in cortical neurons of diabetes mellitus (DM) rats. Original magnification, ×200. (**B** and **C**) Representative gel images and summarized data showing an increase in MAD2B protein expression induced by hyperglycaemia in rat cortices. (**D** and **E**) Representative gel images and summarized data showing the expression of MAD2B in cultured neurons treated with different concentrations of glucose for 24 hrs. (**F**) Representative gel images showing the effect of different concentrations of mannitol on MAD2B protein expression in neurons for 24 hrs. (**G**) Quantitative RT-PCR analysis of MAD2B mRNA level in 50 mM glucose treated neurons. (**H** and **I**) Representative gel image and summarized data showing the effect of hyperglycaemia on MAD2B expression. (**J** and **K**) Representative gel images and summarized data showing the effect of hyperglycaemia on Cdh1 protein expression. Ctrl control; DM diabetes mellitus. *n* ≥ 5 per group. Panel bars display means ± SEM, *P < 0.05 *versus* control.

We first detected that whether hyperglycaemia induced MAD2B expression in STZ-induced DM rat model. Streptozotocin-treated rats exhibited significant increase in blood glucose level and decrease in body weight as we described previously [28]. Results of immunofluorescence staining and Western blot analyses showed that MAD2B was significantly increased in cortical neurons of DM rats (Fig. 1A–C). Then, we further explored whether high glucose directly increased MAD2B expression of *in vitro-*cultured cortical neurons. As shown in Figure 1D and E, different concentrations of glucose markedly increased the expression of MAD2B at 24 hrs in neurons. However, the expression of MAD2B was almost unchanged under different concentrations of mannitol (Fig. 1F), which indicated that the increased expression of MAD2B under high glucose did not result from the changes in osmolality. We chose 50 mM glucose to treat neurons in the following study, because this concentration has been used in many research works to mimic hyperglycaemia *in vitro* [30,31]. It was shown that the expression of MAD2B was increased in a time-dependent manner as detected by real-time RT-PCR (Fig. 1G). MAD2B mRNA expression started to up-regulate after treatment with 50 mM glucose for 12 hrs. Consistent with these data, Western blot (Fig. 1H and I) further showed that MAD2B expression was substantially increased with 50 mM glucose. In the present study, we further detected Cdh1 expression, which is an activator for the anaphase-promoting complex/cyclosome (APC/C). Interestingly, the expression of Cdh1 was almost unchanged as detected by Western blot analyses (Fig. 1J and K).

### Hyperglycaemia induced cyclin B1 accumulation

**Fig. 2:**
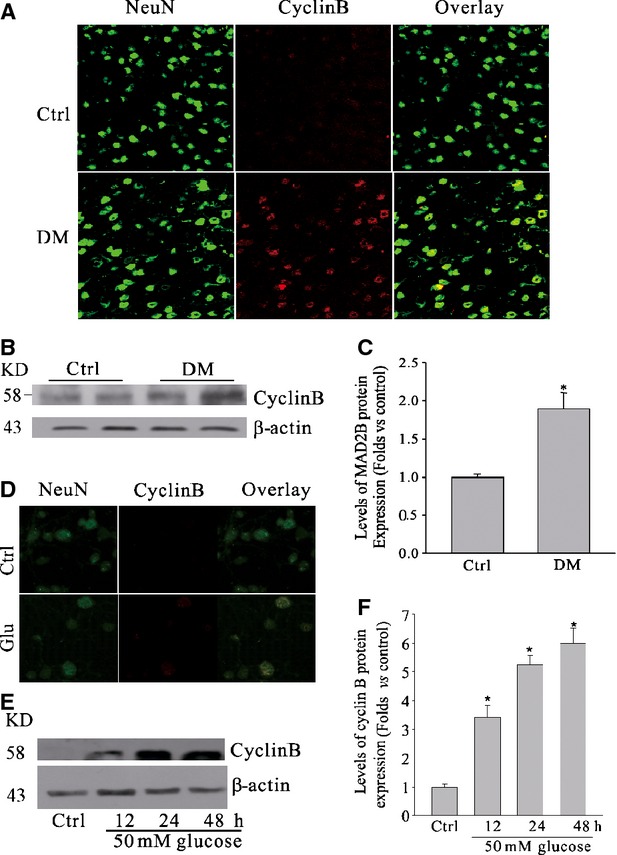
High glucose induced cyclin B1 accumulation in neurons. (**A**) Representative confocal microscopic images of cyclin B1 in cortical neurons of DM rats. (**B** and **C**) Representative gel image and summarized data showing that hyperglycaemia induced an increase of cyclin B1 protein expression in DM rat cortex. (**D**) Representative confocal microscopic images of cyclin B1 cells treated with 50 mM glucose for 24 hrs. (**E** and **F**) Representative gel image and summarized data showing that hyperglycaemia induced an increase of cyclin B1 protein expression in a time-dependent manner. Original magnification, ×200. Images are representative of five batches of cells for each group. NeuN a neuron-specific nuclear protein; Ctrl control; DM diabetes mellitus; Glu Glucose. *n* ≥ 5 per group. Panel bars display means ± SEM, *P < 0.05 *versus* control.

Anaphase-promoting complex/cyclosome activity, regulated by MAD2B, is responsible for cyclin B1 protein stability in post-mitotic neurons [32]. To detect that whether cyclin B1 accumulation in DM rat cortical neurons was changed, we used immunofluorescence staining and Western blot analysis. It was shown that cyclin B1 expression was increased, especially in neurons (Fig. 2A). The expression levels of cyclin B1 were also confirmed by Western blot analyses in cortices of diabetic rats (Fig. 2B and C). Then cyclin B1 protein levels were investigated in hyperglycaemia-treated neurons. Immunofluorescence staining (Fig. 2D) showed that cyclin B1 protein expression was substantially accumulated by treatment of neurons with 50 mM glucose for 24 hrs. Western blot analyses confirmed that hyperglycaemia promoted cyclin B1 accumulation in cultured neurons (Fig. 2E and F).

### MAD2B modulates cyclin B1 stability in cortical neurons

**Fig. 3:**
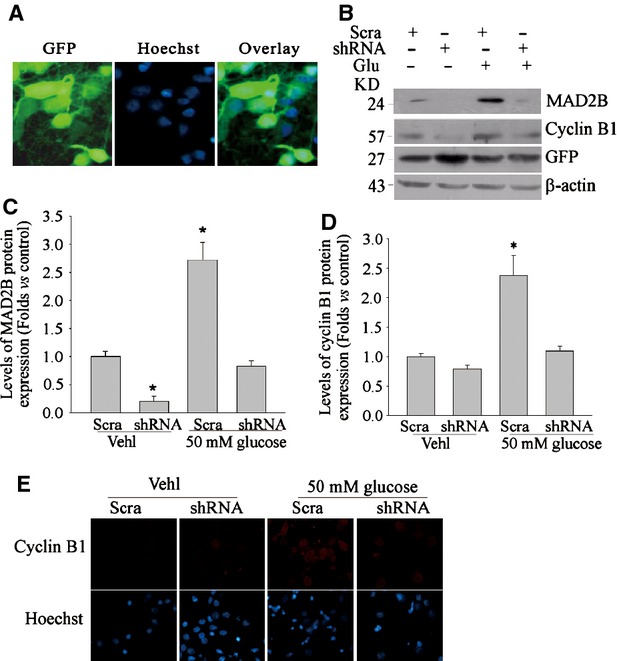
MAD2B modulates cyclin B1 expression in cortical neurons. (**A**) Representative microscopic images showing the infection efficiency of MAD2B shRNA lentivirus; (**B**–**D**) Representative gel images and summarized data showing the effect of MAD2B shRNA on the protein expression of MAD2B and cyclin B1. (**E**) Representative microscopic images showing the effects of MAD2B on accumulation of cyclin B1 in neurons. Green fluorescent protein; shRNA MAD2B shRNA; Scra scrambled shRNA; Glu glucose; Vehl vehicle. *n* ≥ 5 per group. Panel bars display means ± SEM, *P < 0.05 *versus* Scram.

To understand the mechanism responsible for cyclin B1 accumulation in neurons treated with hyperglycaemia, we focused on MAD2B. MAD2B is the recently identified protein in neurons and is an important inhibitor which suppresses APC/C activity. To accomplish this, we developed shRNA lentivirus directed against MAD2B. We first detected the infection efficiency. As shown in Figure 3A, over 70% cells were GFP-positive, which suggested that most cells were infected with lentiviruses. It was shown that infection of primary cortical with these viruses resulted in greater than 80% reduction in MAD2B relative to control viruses as detected by quantitative RT-PCR and Western blot analyses (Fig. 3B and C). Moreover, down-regulation of MAD2B caused a concomitant down-regulation of cyclin B1 accumulation in neurons as detected by immunofluorescence and Western blot analysis, further suggesting MAD2B regulating cyclin B1 stability in neurons (Fig. 3B, D and E).

### Blockade of hyperglycaemia-induced neuronal injury and apoptosis by MAD2B gene silencing

**Fig. 4:**
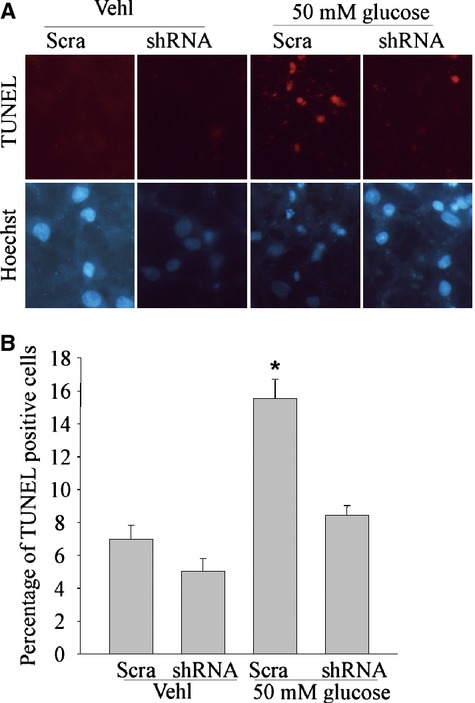
Blockade of high glucose-induced neuronal injury and apoptosis by MAD2B gene silencing. (**A** and **B**) Showing representative microscopic images and summarized data. The results indicated that MAD2B deficiency reduced neuronal apoptosis induced by hyperglycaemia as detected by TUNEL analysis. *Ctrl* control; *Vehl* vehicle; *shRNA* MAD2B shRNA; *Scra* scrambled shRNA. *n* ≥ 5 per group. Panel bars display means ± SEM, *P < 0.05 *versus* Scram.

We hypothesized that changes of MAD2B expression in neurons treated with high glucose might be responsible for neuronal injury and apoptosis. In the present study, we explored the effect of MAD2B expression on neuronal apoptosis. It was demonstrated that hyperglycaemia-induced apoptosis was significantly attenuated by TUNEL analysis in neurons with MAD2B shRNA lentiviruses transfection, compared with scrambled shRNA lentiviruses (Fig. 4A and B).

### Inhibition of MAD2B expression prevents neurons from entering S phase

**Fig. 5:**
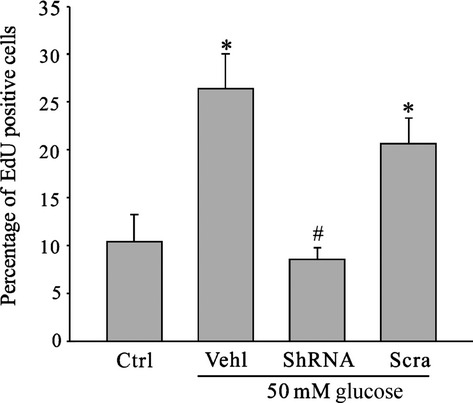
Inhibition of MAD2B expression prevents neurons from entering S phase. Summarized data indicated that MAD2B deficiency reduced the number of cells re-entering S phase induced by high glucose. *Ctrl* control; *Vehl* vehicle; *shRNA* MAD2B shRNA; *Scra* scrambled shRNA. Panel bar displays means ± SEM, *n* ≥ 5 per group. *P < 0.05 *versus* Ctrl, #*P* < 0.05 *versus* Vehl.

It has been shown that APC activity is required to control the progression of dividing cells from G1 into S phase. Therefore, we reasoned that a possible explanation for the cell injury and apoptosis induced by MAD2B expression in neurons is that these post-mitotic cells would attempt to enter S phase before undergoing apoptosis. We analysed the rate of EdU incorporation in hyperglycaemia-treated primary cortical neurons. It was shown that there was an increase in the proportion of cells in S phase in hyperglycaemia-treated neurons when compared with the cells in normal culture media. However, silencing MAD2B reserved the effects of hyperglycaemia on the proportion of cells in S phase (Fig. 5).

## Discussion

The major goal of this study was to determine whether MAD2B, an inhibitor of APC/C activity, is involved in neuronal injury and apoptosis during hyperglycaemia. Using *in vitro* cultured cortical neurons and a STZ-induced DM rat model, we found that hyperglycaemia directly induced expression of MAD2B and cyclin B1 accumulation. Silencing MAD2B expression abolished hyperglycaemia-induced neuronal injury and apoptosis. These results reveal a novel mechanism mediating neuronal injury and apoptosis in DM.

It has been reported that MAD2B can bind indirectly to the APC, thus inhibiting APC activity [33]. The APC/C is a multi-subunit cullin-RING E3 ubiquitin ligase assembled from 13 different core subunits that regulates progression from metaphase to anaphase and exit from mitosis. Several core subunits of APC/C and Cdh1 are highly expressed in mammalian brain post-mitotic neurons [34]. The activity of the APC/C is tightly regulated along the cell cycle. To be active, the APC/C requires the binding of either one of two WD40-domain co-activator proteins, Cdc20 or Cdh1, which also participate in substrate recognition [35,36]. Interestingly, although the APC regulatory subunit Cdc20 does not appear to be expressed in the adult brain, Cdh1 is highly expressed in neurons in many parts of the CNS, including the cerebral cortex, hippocampus and cerebellum. Anaphase-promoting complex/cyclosome is active from the onset of mitosis to the end of the G1 phase of the cell cycle. Differentiated cells, such as neurons, remain resting in the G0 phase because of an active down-regulation of cell cycle-related proteins.

Recent evidence has indicated that neurons retain the ability to reactivate the cell cycle in response to CNS insults. It has been shown that APC/C plays a vital role in post-mitotic neurons. Most researchers focus on the roles of APC activators in neurons. However, few studies showed the expression of the inhibitors of APC such as MAD2 and MAD2B in neurons. In the present study, we used the DM rat model to detect the expression of MAD2B in cortex. It was shown that expression of MAD2B was highly increased in DM rats compared with control rats. Then we used primary cultured neurons to explore the direct role of high glucose on MAD2B expression. We found that MAD2B expression was increased in a time-dependent manner under high glucose. Moreover, we detected the expression of Cdh1 expression, one of the major APC activators in neurons. It was shown that levels of Cdh1 protein expression were not changed. This suggested that MAD2B might play an important role in regulating APC activity under high glucose, and thus mediate the neuronal injury under hyperglycaemia.

In different cells, there are different substrates for APC. In muscle, APC/C-Cdh1 drives cell differentiation through the destruction of two proteins, Skp2 and Myf5 [37]. In lens, APC/C-Cdh1 facilitates SnoN degradation and then regulates lens differentiation [38]. In neurons, the APC/C-Cdh1 complex actively down-regulates the stability of the cell-cycle protein cyclin B1. Keeping cyclin B1 destabilized is critical for preventing the aberrant re-entry of post-mitotic neurons into the cell cycle [32,39,40]. Cyclin B1 belongs to the cyclin family including cyclin A, B, D, E and others. Cyclins provide the crucial hint about the chemical mechanism of the cell-cycle oscillator and the important role for regulated proteolysis in eukaryotes. In cell cycle, B-type cyclins play pivotal roles in regulating the G2/M transition and in M-phase progression [41]. Three B-type cyclins, B1, B2 and B3, have been identified in mammals [42]. Among the three B-type cyclins, Cyclin B1 is well studied. It has been found that cyclin B1-null mice die *in utero* and no homozygous B1-null pups were born, which suggest that cyclin B1 is an essential gene in development [43]. In the CNS, it was found that aberrant expression of cyclin B1 with other cell-cycle proteins preceded neuronal death after chronic cerebral hypoperfusion in rats [44]. Significant differences in cyclin B were also found when comparing senescence-accelerated mice 8 with control strain [45]. Stimulation of *N*-methyl-d-aspartate receptors that occurs in neurodegenerative diseases promoted the accumulation of cyclin B1 in the nuclei of cortical neurons, which led the neurons to undergo apoptotic death [40]. All these data suggested that cyclin B1 plays an important role in neuronal survival. In the present study, we found that MAD2B was increased under hyperglycaemia *in vivo* and *in vitro*. However, whether cyclin B1, one substate of APC, is involved in hyperglycaemia-induced cellular injury is still unknown. In the present study we detected the expression of cyclin B1 under hyperglycaemia *in vitro* neurons. It was shown that levels of cyclin B1 were elevated in a time-dependent manner as MAD2B. Like the expression of MAD2B in cortex, cyclin B1 expression was also increased in cortex of DM rats. These results strongly suggested that elevated expression of MAD2B induced by hyperglycaemia results in cyclin B1 accumulation in neurons.

To further support this view, we used a shRNA specifically targeted against MAD2B in primary cultured neurons. In the present study, it was found that the hyperglycaemia-induced expression of MAD2B was highly inhibited with the shRNA of MAD2B, indicating a successful establishment of cellular model to explore the role of MAD2B in neurons. Interestingly, in neurons with MAD2B shRNA, the expression of cyclin B1 induced by high glucose was also significantly reduced, implying that MAD2B deficiency prevents hyperglycaemia-induced cyclin B1 expression. One of the most important findings of the present study is that MAD2B deficiency may protect neurons from hyperglycaemia-induced neuron injury, which was supported by attenuated cell apoptosis detected by TUNEL analysis. These results were in consistent with those of a previous study, which indicated that the inhibition of cyclin B1 accumulation significantly increased the survival of Cdh1-depleted neurons. In our study, it was shown that MAD2B shRNA incompletely inhibited cyclin B1 expression and MAD2B shRNA completely inhibited glucose-induced apoptosis, which seemed paradoxical. We deduced that MAD2B mediated neuron injury induced by hyperglycaemia mainly through regulating cyclin B1. Whether there are novel substrates involved in this process needs to be further elucidated in our future study. In concert, the results from these *in vitro* experiments further support the view that MAD2B is critically involved in mediating cyclin B1 accumulation and consequent neuron impairment.

It has been shown that silencing of Cdh1, APC activator, in both (SH-SY5Y) cells previously subjected to *in vitro* differentiation and post-mitotic cortical neurons significantly promoted entry into S phase that would be lethal in terminally differentiated cells [34]. Moreover, concomitant depletion of cyclin B1 prevented neurons from re-entering the cell cycle and rescued the cell-death phenotype S phase and mitosis. To detect whether hyperglycaemia induced neurons re-entering the cell cycle and the role of MAD2B, we detected the S-phase cells. It was shown that MAD2B deficiency highly decreased the percentage of S-phase cells. The possible mechanism may be that hyperglycaemia-induced MAD2B expression leads to cyclin B1 accumulation, and then resulting in neuron re-entering S phase and apoptosis. As regards how cyclin B1 triggers neurodegeneration, there are two major explanations. One is that Cdk1/cyclin B1 activity could mediate the cell death by catalysing BAD phosphorylation [46]. The other is that cyclin B1 could directly trigger entry into S phase and mitosis.

In summary, we demonstrated that hyperglycaemia induced the expression of MAD2B, which resulted in cyclin B1 accumulation. The amelioration of cellular injury in MAD2B deficiency neurons further strengthened the conclusion that MAD2B plays an important role in mediating hyperglycaemia-induced neuron apoptosis. Although the mechanisms which underlie the increased expression of MAD2B under hyperglycaemia are still unknown, the present study for the first time demonstrates that MAD2B contributes to neurons undergoing apoptosis *via* APC-cyclin B1 pathway, suggesting a new pathogenic pathway governing cellular responses to hyperglycaemia, which may provide an important therapeutic strategy to prevent neuron injury in diabetic encephalopathy.
